# Combined assessment of progressive apraxia of speech brain microstructure by diffusion tensor imaging tractography and multishell neurite orientation dispersion and density imaging

**DOI:** 10.1002/brb3.3346

**Published:** 2024-01-02

**Authors:** Rodolfo G. Gatto, Gabriela Meade, Joseph R. Duffy, Heather M. Clark, Rene L. Utianski, Hugo Botha, Mary M. Machulda, Keith A. Josephs, Jennifer L. Whitwell

**Affiliations:** ^1^ Department of Neurology Mayo Clinic Rochester Minnesota USA; ^2^ Department of Psychiatry and Psychology Mayo Clinic Rochester Minnesota USA; ^3^ Department of Radiology Mayo Clinic Rochester Minnesota USA

**Keywords:** 4R tauopathies, diffusion tensor imaging, neurite orientation dispersion density imaging, progressive apraxia of speech, tractography

## Abstract

**Background:**

Progressive apraxia of speech (PAOS) is characterized by difficulties with motor speech programming and planning. PAOS targets gray matter (GM) and white matter (WM) microstructure that can be assessed using diffusion tensor imaging (DTI) and multishell applications, such as neurite orientation dispersion and density imaging (NODDI). In this study, we aimed to apply DTI and NODDI to add further insight into PAOS tissue microstructure.

**Methods:**

Twenty‐two PAOS patients and 26 age‐ and sex‐matched controls, recruited by the Neurodegenerative Research Group (NRG) at Mayo Clinic, underwent diffusion MRI on 3T MRI. Brain maps of fractional anisotropy (FA) and mean diffusivity (MD) from DTI and intracellular volume fraction (ICVF) and isotropic volume fraction (IsoVF) from NODDI were generated. Global WM and GM, and specific WM tracts were identified using tractography and lobar GM regions.

**Results:**

Global WM differences between PAOS and controls were greatest for ICVF, and global GM differences were greatest for MD and IsoVF. Abnormalities in key WM tracts involved in PAOS, including the body of the corpus callosum and frontal aslant tract, were identified with FA, MD, and ICVF, with excellent differentiation of PAOS from controls (area under the receiver operating characteristic curves >.90). MD and ICVF identified abnormalities in arcuate fasciculus, thalamic radiations, and corticostriatal tracts. Significant correlations were identified between an index of articulatory errors and DTI and NODDI metrics from the arcuate fasciculus, frontal aslant tract, and inferior longitudinal fasciculus.

**Conclusions:**

DTI and NODDI represent different aspects of brain tissue microstructure, increasing the number of potential biomarkers for PAOS.

## Graphical abstract

White matter and gray matter segmented regions from intracellular and isotropic volume fraction maps in a patient with progressive apraxia of speech.

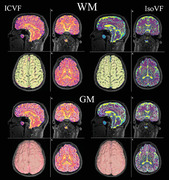

## 1 INTRODUCTION

Progressive apraxia of speech (PAOS), part of a broader group of diseases categorized as frontotemporal lobar degeneration, is a motor speech disorder characterized by slow speaking rate, distorted sound substitutions, additions, repetitions, and prolongations, segmentations between syllables and words, as well as trial‐and‐error articulatory movements (Duffy, 2006; Josephs et al., 2021; Jung et al., 2013). More specifically, PAOS generally represents a compromised ability to plan or program speech movements resulting in phonetically and prosodically atypical speech (Duffy et al., 2021). Patients who present with apraxia of speech (AOS) in isolation are diagnosed with primary PAOS (Josephs et al., 2012). Others also develop language difficulties in the form of agrammatic aphasia (Josephs et al., 2013), but their AOS often remains the predominant problem. The term PAOS is as an umbrella term that includes both patients with and without an agrammatic aphasia (Duffy et al., 2021; Valls Carbo et al., 2022). Patients with an agrammatic aphasia could also be diagnosed as nonfluent/agrammatic primary progressive aphasia if language deficits are the dominant feature (Gorno‐Tempini et al., 2011).

Patients with PAOS show gray matter (GM) atrophy in the supplementary motor area (SMA) and lateral premotor cortex (Josephs et al., 2013, 2012), with involvement of Broca's area particularly in those with coexisting agrammatic aphasia, that is, progressive agrammatic/nonfluent aphasia (AOS‐PAA) (Gorno‐Tempini et al., 2004; Josephs et al., 2013). White matter (WM) degeneration has also been demonstrated on diffusion tensor imaging (DTI) and diffusion tractography, with degeneration of the superior longitudinal fasciculus, the body of the corpus callosum, frontal aslant tract, and other WM tracts projecting to and from the SMA (Botha et al., 2015; Josephs et al., 2013, 2018; Valls Carbo et al., 2022). The severity of AOS correlates with the integrity of the SMA commissural fibers, while the severity of agrammatic aphasia correlates with the integrity of the frontal aslant tract; hence, DTI metrics mirror clinical impairment in PAOS (Valls Carbo et al., 2022).

However, the use of DTI has intrinsic limitations. As an example, DTI has a limited resolution to capture crossing and curving areas, and its measures are limited to the study of non‐Gaussian hindered compartments (Jones, 2010; Kierońska & Słoniewski, 2019). To address these issues, additional diffusion techniques, such as neurite orientation dispersion and density imaging (NODDI), have been proposed as an alternative and improved diffusion model in the interrogation of brain tissue in the context of different neurodegenerative diseases (Gatto et al., 2019; Kamagata et al., 2016; Kamiya et al., 2020; Zhang et al., 2012). NODDI probes the microstructure of neurites (i.e., axons and dendrites) by modeling three components of the brain tissue. Three metrics are produced that quantify the fractional volume of intracellular neurite density or intracellular volume fraction (ICVF), intracellular neurite dispersion or orientation dispersion index (ODI), and extracellular fluid or isotropic volume fraction (IsoVF) As such, it has been noted that NODDI could provide biologically meaningful metrics that have been linked with histological measures in neural tissue (Gatto et al., 2018). A study carried out by Raghavan et al. used NODDI as a multicompartmental model to study the vascular WM component located at the genu of the corpus callosum region in subjects with atypical dementia with a mean age of 68.3 ± 13.1 years. They found that ICVF and IsoVF were sensitive imaging biomarkers to represent microstructural changes. As such, advanced diffusion models may add significant value to separate the underlying cause of cognitive impairment in microvascular disease, tau protein deposition, or TDP‑43 pathologies (Raghavan et al., 2022). Therefore, investigating the potential of NODDI in clinical studies could greatly increase our understanding of PAOS brain pathology.

To the best of our knowledge, the combined exploratory use of DTI and NODDI has not been yet applied to specifically characterize brain microstructural changes in patients with PAOS. In this study, we hypothesized that NODDI, compared to results from DTI, will capture larger, not otherwise captured, microstructural changes in WM and GM in patients with PAOS.

## 2 MATERIAL AND METHODS

### 2.1 Participants

From July 2018 to May 2022, a total of 23 patients with PAOS, recruited by the Neurodegenerative Research Group at Mayo Clinic, Rochester, MN, underwent detailed speech and language and neurological evaluations and 3T MRI. One subject from the PAOS group was removed from analysis due to significant brain morphological changes that made it impossible to perform automatic segmentation or reliable tracking approaches. From these PAOS subjects, nine (39% of total) were initially diagnosed with primary PAOS (no more than equivocal evidence of aphasia). The remaining 14 patients (68% of total) had AOS and a progressive agrammatic aphasia. This cohort does not overlap with the cohort of patients published in our previous tractography study (Valls Carbo et al., 2022). A group of 26 age‐ and sex‐matched healthy controls was also recruited. Controls were included if they did not have any reported cognitive, motor, or behavioral abnormalities and performed normally on the Montreal Cognitive Assessment (MoCA) Battery (≥26) and the Hoehn and Yahr scale (score of 0). The study was approved by the Mayo Clinic IRB committee, and informed consent from the patient or direct relatives was obtained from all participants.

### 2.2 Clinical evaluations

The speech and language evaluations were administered by one of three board‐certified speech–language pathologists (JRD, HMC, RLU) and included assessments targeting motor speech and language functions. To assess for AOS, the Apraxia of Speech Rating Scale‐3 (ASRS‐3) (Clark et al., 2021; Duffy et al., 2023; Strand et al., 2014) and the Articulatory Error Score (AES) assessment (Utianski et al., 2021) were used. The ASRS‐3 is an index of the presence and severity of 13 speech features associated with AOS, where higher scores reflect greater severity. We have recently shown that the ASRS‐3 is a reliable and valid scale for identifying the presence and severity of AOS and its predominant features, and it has excellent sensitivity to AOS presence and outstanding specificity relative to aphasia and dysarthria in patients with neurodegenerative disease (Duffy et al., 2023). The AES requires patients to repeat multisyllabic words and sentences and indexes the percentage of words on which they make sound‐level errors (e.g., distorted substitutions, omissions, prolongations) (Utianski & Josephs, 2023; Utianski et al., 2018). The presence of dysarthria was assessed using the same speech tasks and corroborated by oral mechanism findings. The determination of a co‐occurring agrammatic aphasia was made based on comprehensive language testing that included the Western Aphasia Battery—Revised (Kertesz, 2006), including supplementary writing tasks, a 15‐item Boston Naming Test (Lansing et al., 1999), the 22‐item version of the Token Test (De Renzi & Vignolo, 1962), and syntactic subtests from the Boston Diagnostic Aphasia Examination—Third Edition (Goodglass et al., 2001). To be diagnosed with AOS‐PAA, patients needed to demonstrate impairment on at least two measures of grammar (i.e., spoken and written picture description, syntactic subtests from the Boston Diagnostic Aphasia Examination). Here, we present results from the Western Aphasia Battery (WAB) for all patients as a global measure of language function. Correlation analyses were also performed with lexical fluency measures, which included the animal fluency subtest of the WAB (i.e., name as many animals as you can in 1 min) and a total letter fluency score (i.e., name as many words as you can think of that start with F‐, A‐, and S‐, with 1 min per letter). The neurologic testing was conducted by a behavioral neurologist (KAJ, HB) and included assessments to characterize general cognitive ability, such as the MoCA (Nasreddine et al., 2005). Parkinsonism was assessed by the Movement Disorders Society‐sponsored version of the Unified Parkinson's Disease Rating Scale motor subsection (MDS‐UPDRS III) (Goetz et al., 2008), and ideomotor apraxia was assessed by the praxis subtest of the WAB (Kertesz, 2022).

### 2.3 Image acquisition and preprocessing

Patients underwent diffusion‐weighted MRI with a 3T Siemens scanner. The imaging protocol included a three‐dimensional Magnetization Prepared Rapid Acquisition Gradient‐Echo (MPRAGE) sequence as well as a diffusion magnetic resonance imaging (dMRI) sequence with a spin‐echo single‐shot echo planar imaging (EPI) sequence. The time to echo (TE) was set at 71 ms, and the repetition time (TR) was set at 3400 ms. Each dMRI scan included a multishell set of four diffusion‐weighted values: *b* = 0, *b* = 500 s/mm^2^, *b* = 1000 s/mm^2^, and *b* = 2000 s/mm^2^. The number of diffusion sampling directions was 6, 48, and 60, respectively. The in‐plane resolution and slice thickness for each section were originally obtained at 2 mm and resampled to 0.5 mm. Images were corrected for head motion, Eddy current, and susceptibility artifact distortions using standard algorithms extensively described in the literature (Andersson et al., 2018). The *b*‐table was checked by an automatic quality control routine to ensure its accuracy (Schilling et al., 2019). The accuracy and reliability of the diffusion data were also checked by incorporated algorithms (Yeh et al., 2019). NODDI calculations were performed using the qMRILab toolbox version 2.4.1 incorporated and run in MATLAB (Karakuzu et al., 2020). Output parameters such as ICVF and IsoVF were collected as NIfTI files.

### 2.4 DTI calculation and tractography reconstructions

The number of seed points was manually selected based on a balance given by input data and an optimal number of the tracts obtained during visual inspections. Previously described criteria were applied for selecting the number of optimal seed points (Cheng et al., 2012; Zajac et al., 2017). An average of 500,000 seeds were placed with a distance voxel tolerance of 16 mm. Distorted tract or false connections were automatically removed using a topology‐informed pruning algorithm (Yeh et al., 2019). Regions where fractional anisotropy (FA) was below 0.2 were automatically removed as they were unable to track. Whole‐brain deterministic fiber tracking algorithms with augmented tracking strategies to improve reproducibility were employed (Yeh et al., 2013). Anisotropy and angular threshold, as well as step size, were automatically selected. An anatomical tractography atlas was used to map all tracts, and seeding regions were placed using preestablished WM probabilistic areas (Yeh et al., 2018). Tracks with a length shorter than 3 mm or longer than 500 mm were discarded. WM tack reconstructions were determined by a topographical anatomical atlas embedded in the DSI software.

### 2.5 Region of interest analysis

**FIGURE 1:**
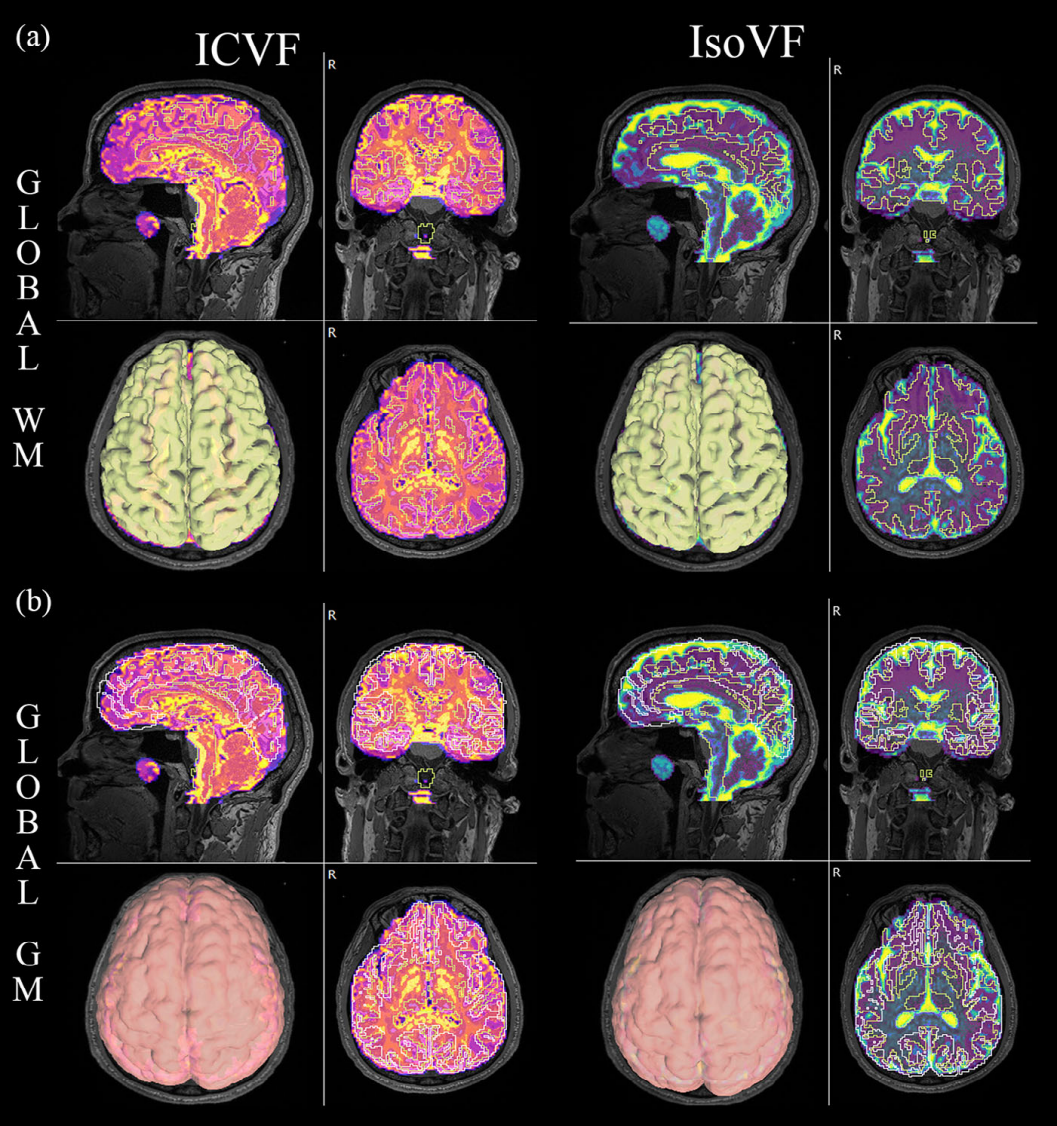
Segmented global white matter (WM) and gray matter (GM) regions from progressive apraxia of speech (PAOS) patient. (A) Segmented whole‐brain WM regions from intracellular volume fraction (ICVF) and isotropic volume fraction (IsoVF) brain maps. (B) GM segmentations on ICVF and iso‐VF maps.

**FIGURE 2:**
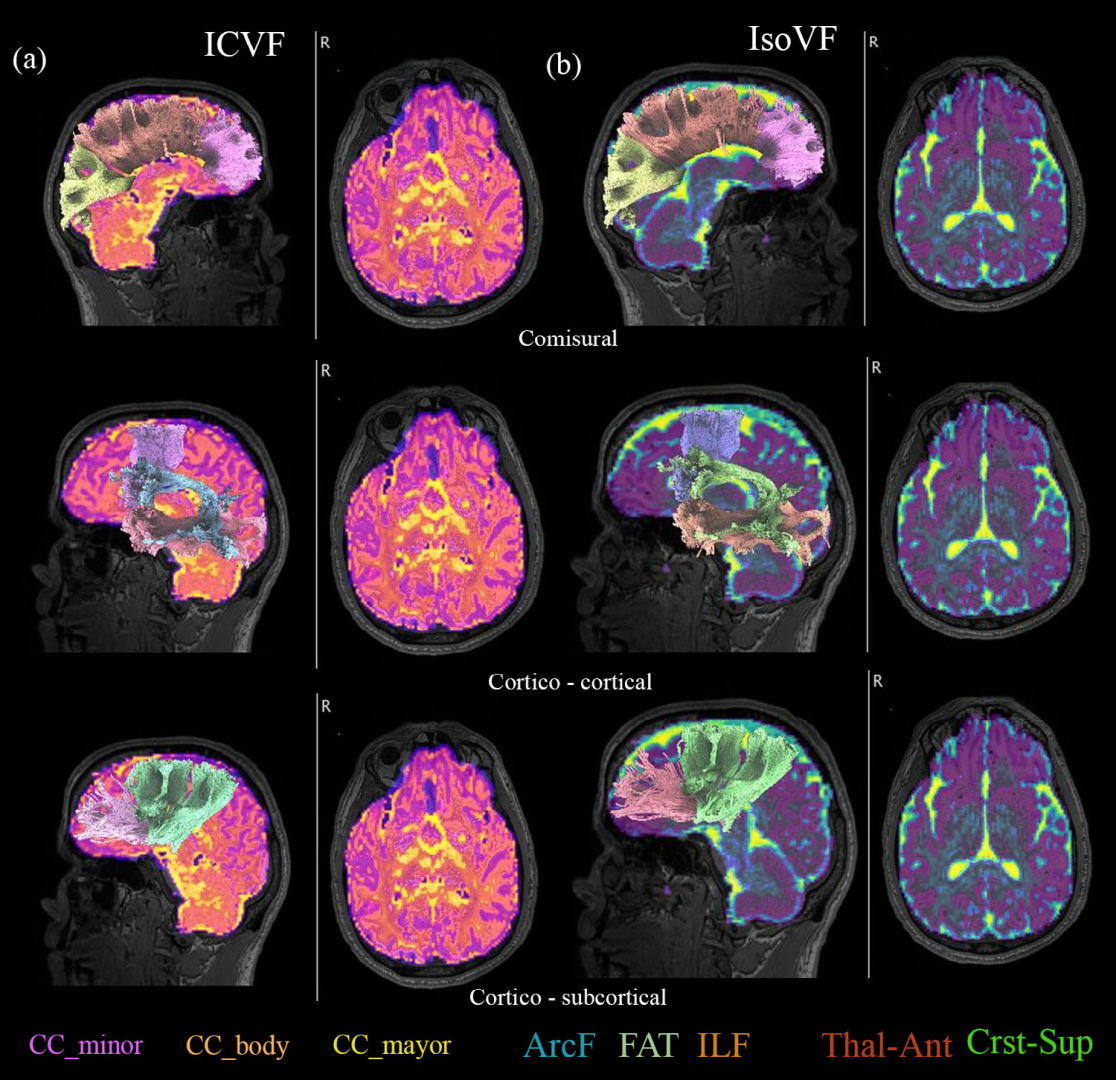
Coregistration of neurite orientation dispersion and density imaging (NODDI) brain maps and diffusion tensor imaging (DTI)‐based tractography. (A) Fractography projection on intracellular volume fraction (ICVF) maps on a patient with progressive apraxia of speech (PAOS). Three different segments from the corpus callosum (CC) were reconstructed (cc‐minor, cc‐body, and cc‐mayor). Additional transcortical tracts such as the arcuate fasciculus (AF) and frontal aslant tracts (FAT) as well as inferior longitudinal fasciculus (ILF) were considered. (B) Isotropic volume fraction (IsoVF) maps are displayed with previously described tracts as well as anterior thalamic radiations (Thal_ant.) and corticostriatal anterior (Cst_sup).

**FIGURE 3:**
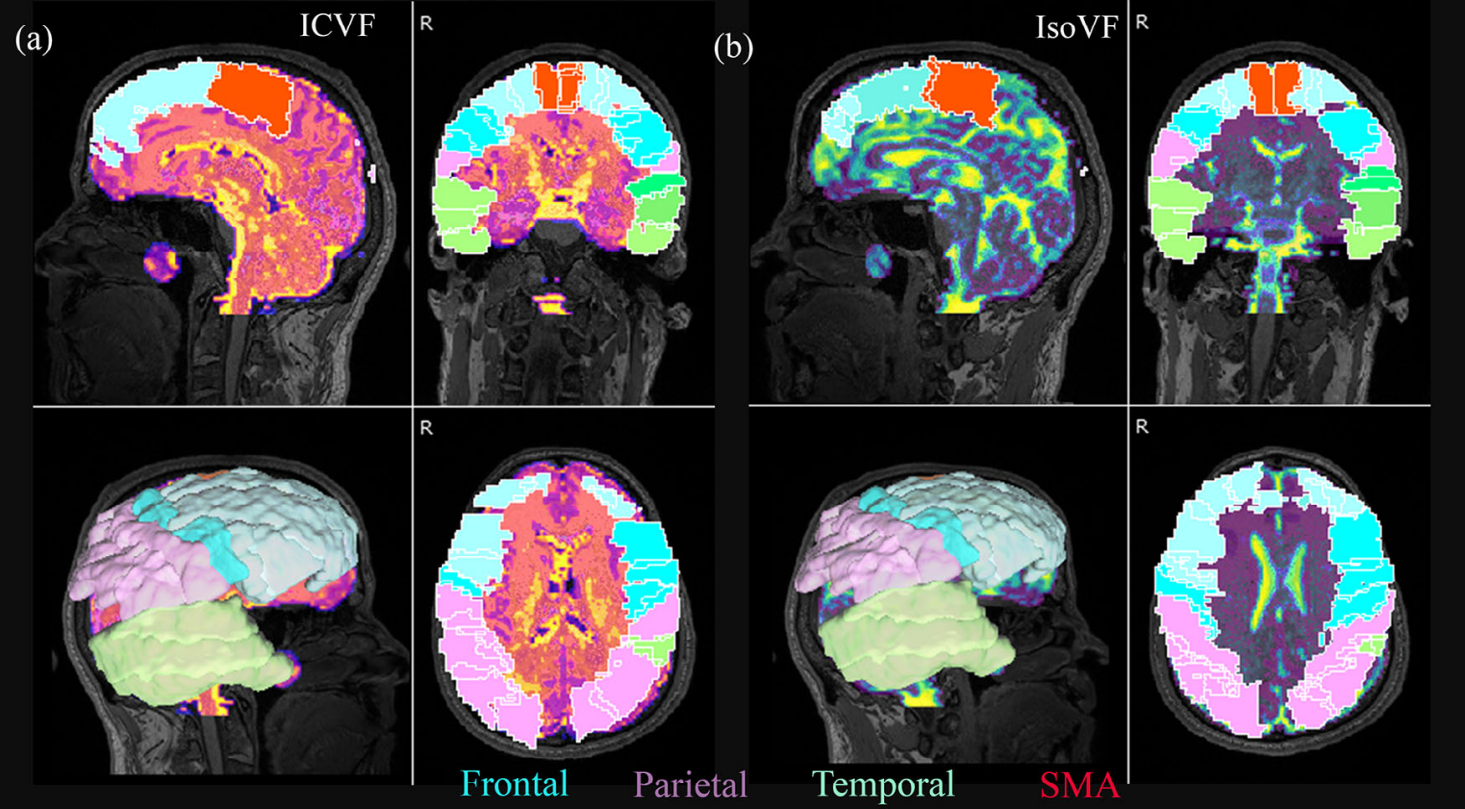
Automated anatomical labeling atlas 2 (AAL2)‐based gray matter (GM) segmentation on individual space from a progressive apraxia of speech (PAOS) patient. Each GM region of interest (ROI) is projected on an intracellular volume fraction (ICVF) (A) and isotropic volume fraction (IsoVF) maps (B). GM subregions were combined into three main GM regions: (1) a frontal region, from a combination of seven subregions—frontal inferior operculum, frontal inferior pars triangularis, frontal middle gyrus, frontal superior gyrus, frontal superior medial gyrus, precentral gyrus, and supplementary motor area (SMA) (blue); (2) a temporal region, combining three subregions—temporal inferior, temporal middle, and temporal superior gyrus (green); and (3) a parietal region, which combines four subregions—parietal inferior, parietal superior, supramarginal, and postcentral gyrus (pink). The right and left areas were analyzed separately. Note that although the SMA has been included in the frontal regions (red), it was also analyzed separately.

Three main groups of WM tracts were interrogated: (1) commissural tracts, such as the minor, body, and major portion of the corpus callosum (CC) body; (2) right and left corticocortical tracts, such as the arcuate fasciculus (ArcF), the frontal aslant tract (FAT), and the inferior longitudinal fasciculus (ILF), and right and left corticosubcortical tracts; and (3) inner portion of the superior longitudinal fasciculus (SLF‐3), the thalamic anterior radiation (Thal_Ant), as well as the corticostriatal superior tract (Cstr_Sup). Therefore, a total of 26 WM tracts were reconstructed per participant (Figure 1). Segmentation of the brain's GM was established using the AAL2 atlas (Rolls et al., 2015) (Figure 2). We assessed three main areas: (1) a frontal region, constructed from a combination of seven subregions—frontal inferior operculum, frontal inferior pars triangularis, frontal middle gyrus, frontal superior gyrus, frontal superior medial gyrus, precentral gyrus, and SMA; (2) a temporal region constructed from three subregions—temporal inferior, temporal middle, and temporal superior gyrus; and (3) a parietal region combining four subregions—parietal inferior, parietal superior, supramarginal, and postcentral gyrus. Each region was independently considered in the right and left hemisphere for a total of six GM regions per participant. Due to its significant role in PAOS, SMA regions were also independently considered for the analysis (Figure 3).

### 2.6 Statistical analysis

PAOS and control groups’ descriptive demographics were compared using nonparametric Mann–Whitney statistical tests. The *p*‐values are from Wilcoxon rank‐sum tests or Fisher's exact tests where appropriate (see Table 1). For DTI and NODDI parameters, Mann–Whitney statistical tests and two‐tailed calculations were used where appropriate. A *p*‐value of <.05 was defined as statistically significant. Benjamini–Hochberg false discovery rate (FDR) correction was used to account for multiple comparisons across multiple WM tracts and GM ROIs. FDR <.05 was deemed significant. An area under the receiver operating characteristic curve (AUROC) analysis was performed to assess the ability of metrics to differentiate PAOS from controls. Correlative comparisons between dMRI‐derived parameters, such as FA, mean diffusivity (MD), ICVF, and Iso‐VF, and clinical assessments (e.g., percentage of errors on the AES, verbal fluency) were measured using Spearman's correlation coefficient (*ρ*).

**TABLE 1:** Participant demographics.

	PAOS (*n* = 22)	Control (*n* = 26)	*p*‐values
Female, *n* (%)	14 (63%)	13 (50%)	.39
Handedness, *n* (%)			>.99
Left	3 (14%)	4 (16%)	
Right	19 (86%)	22 (84%)	
Education, years	16 (14, 18)	16 (14, 19)	.87
Age at visit, years	66 (60, 73)	63 (56, 71)	.12
Age at onset, years	62 (55, 71)		
Time from onset to visit, years	3.2 (1.7, 4.8)		
MoCA/30	23 (20, 27)	27 (26, 28)	<.0002
WAB‐AQ/100	91 (84, 98)		
ASRS‐3/52	23 (18, 26)		
AES (tt_pct_err)/100	32 (13, 49)		
MDS‐UPDRS III/132	12 (7, 15)		
WAB praxis/60	56 (54, 59)		
Letters of fluency (sum)	8 (4, 11)		
WAB animal fluency	13 (10, 17)		

*Note*: The *p*‐values are from the Wilcoxon rank‐sum test or Fisher's exact test where appropriate. Data shown are median (interquartile range) or *n* (%).

Abbreviations: AES, Articulatory Error Score; ASRS, Apraxia of Speech Rating Scale; MDS‐UPDRS, Movement Disorder Society‐Sponsored Revision of the Unified Parkinson's Disease Rating Scale; MoCA, Montreal Cognitive Assessment; PAOS, progressive apraxia of speech; tt_pct_err, total percentage of error; WAB‐AQ, Western Aphasia Battery—Aphasia Quotient.

## 3 RESULTS

### 3.1 Participant characteristics

No significant differences were observed in sex, education, handedness, or age at first visit between PAOS and controls (Table 1). However, there were significant differences in general cognition as assessed by the MoCA, as expected (*p* < .0002). Quantitative characteristics of different speech and language assessments for the PAOS group are also presented in Table 1. Fourteen patients were judged to have a co‐occurring agrammatic aphasia and four of those patients also had dysarthria (*n* = 3 spastic, *n* = 1 hypokinetic).

### 3.2 ROI analysis

**FIGURE 4:**
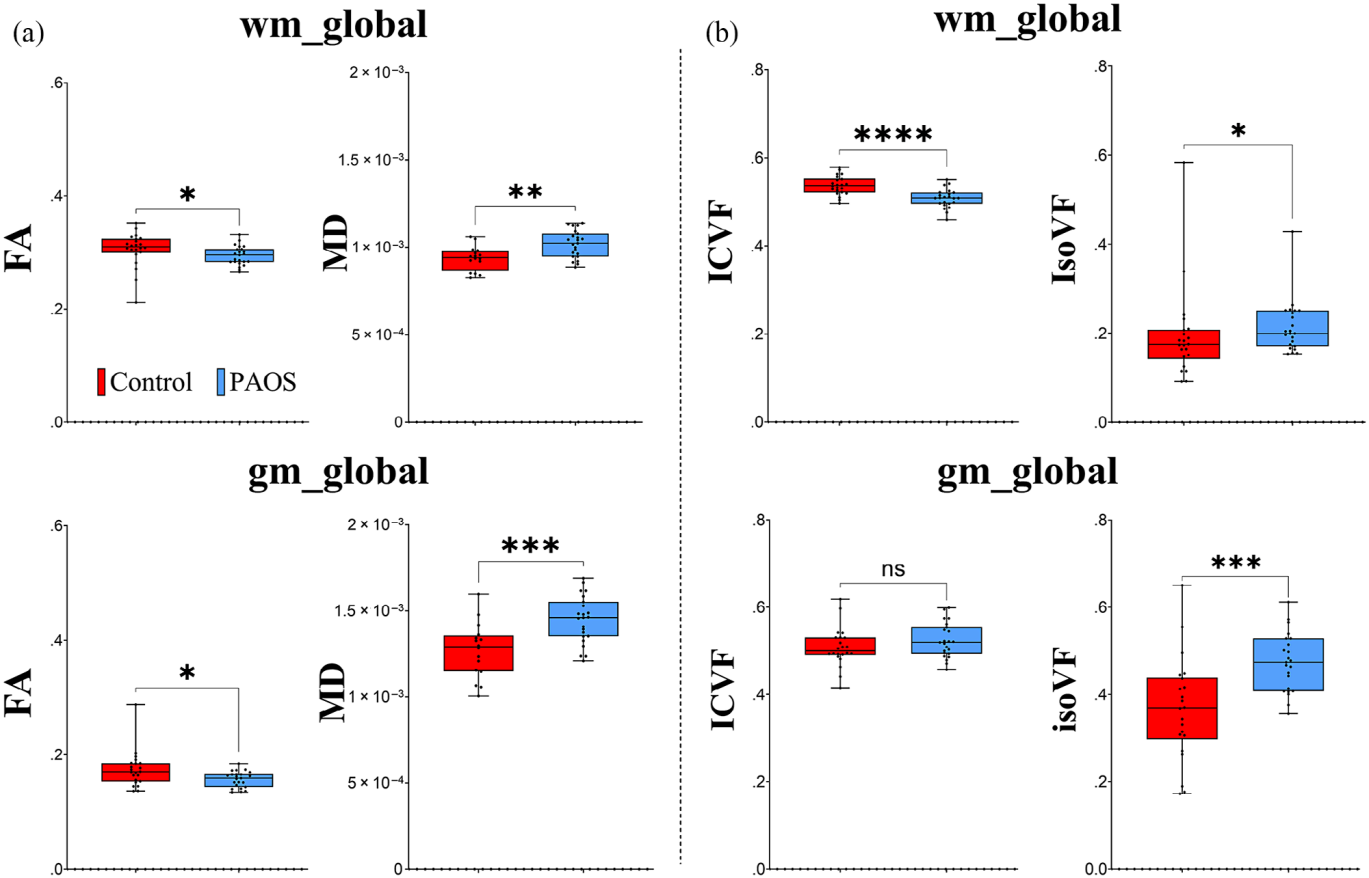
Global white matter (WM) and gray matter (GM) analysis obtained from diffusion tensor imaging (DTI) and neurite orientation dispersion and density imaging (NODDI) parameters. (A) DTI analysis using fractional anisotropy (FA) and mean diffusivity (MD) outputs demonstrated a significant decrease in FA and an increase in MD in WM and GM regions from progressive apraxia of speech (PAOS) patients. Larger differences were observed in MD in the PAOS group (GM > WM). (B) NODDI calculation has shown a significant decrease in intracellular volume fraction (ICVF) in the PAOS group but not significant in whole‐brain GM (gm_global). Isotropic volume fraction (IsoVF) was significantly increased in whole‐brain WM (wm_global) (*p* < .05) and gm_global (*p* < .001) in PAOS patients. *p < .05; **p < .01; ***p < .001.

**FIGURE 5:**
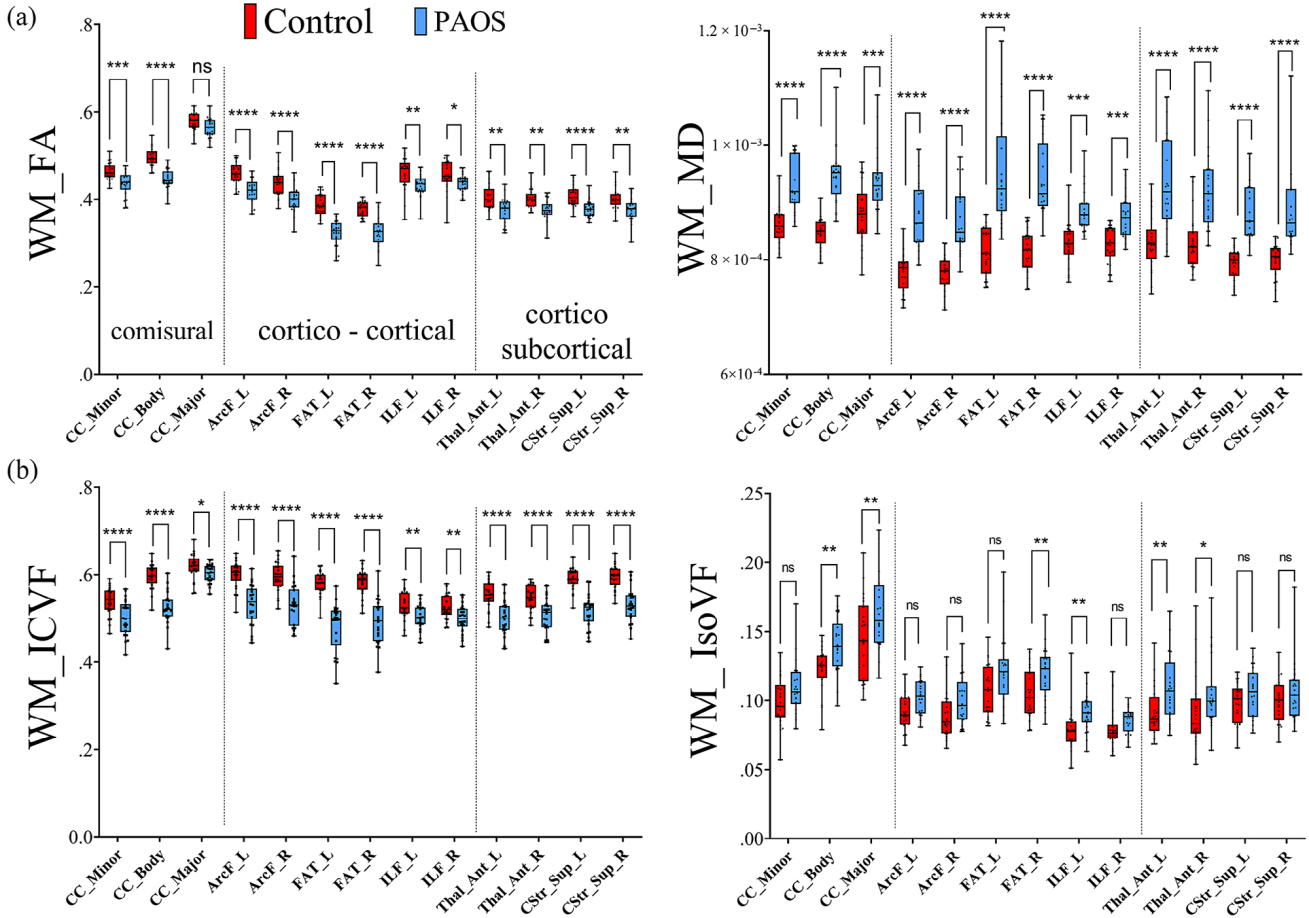
Diffusion tensor imaging (DTI) outputs extracted from tractography reconstructions and neurite orientation dispersion and density imaging (NODDI) region of interest (ROI) projections. Three main groups of tracts were interrogated: (1) commissural tracts, such as the minor, body, and major portion of the corpus callosum (CC); (2) right and left corticocortical tracts, such as the arcuate fasciculus (ArcF), the frontal aslant tract (FAT), and the inferior longitudinal fasciculus (ILF); and (3) right and left corticosubcortical tracts, including the thalamic anterior radiation (Thal_Ant) as well as the corticostriatal superior tract (Cstr_Sup). (A) Fractional anisotropy (FA) and mean diffusivity (MD) from white matter (WM) tracts are analyzed. There was a significantly larger difference between controls and progressive apraxia of speech (PAOS) groups in MD measurements across commissural, corticocortical, and corticosubcortical tracts. (B) NODDI WM tract ROI projections demonstrate a significantly larger difference between groups in intracellular volume fraction (ICVF) compared to isotropic volume fraction (IsoVF) in all three groups of WM tracts.

**FIGURE 6:**
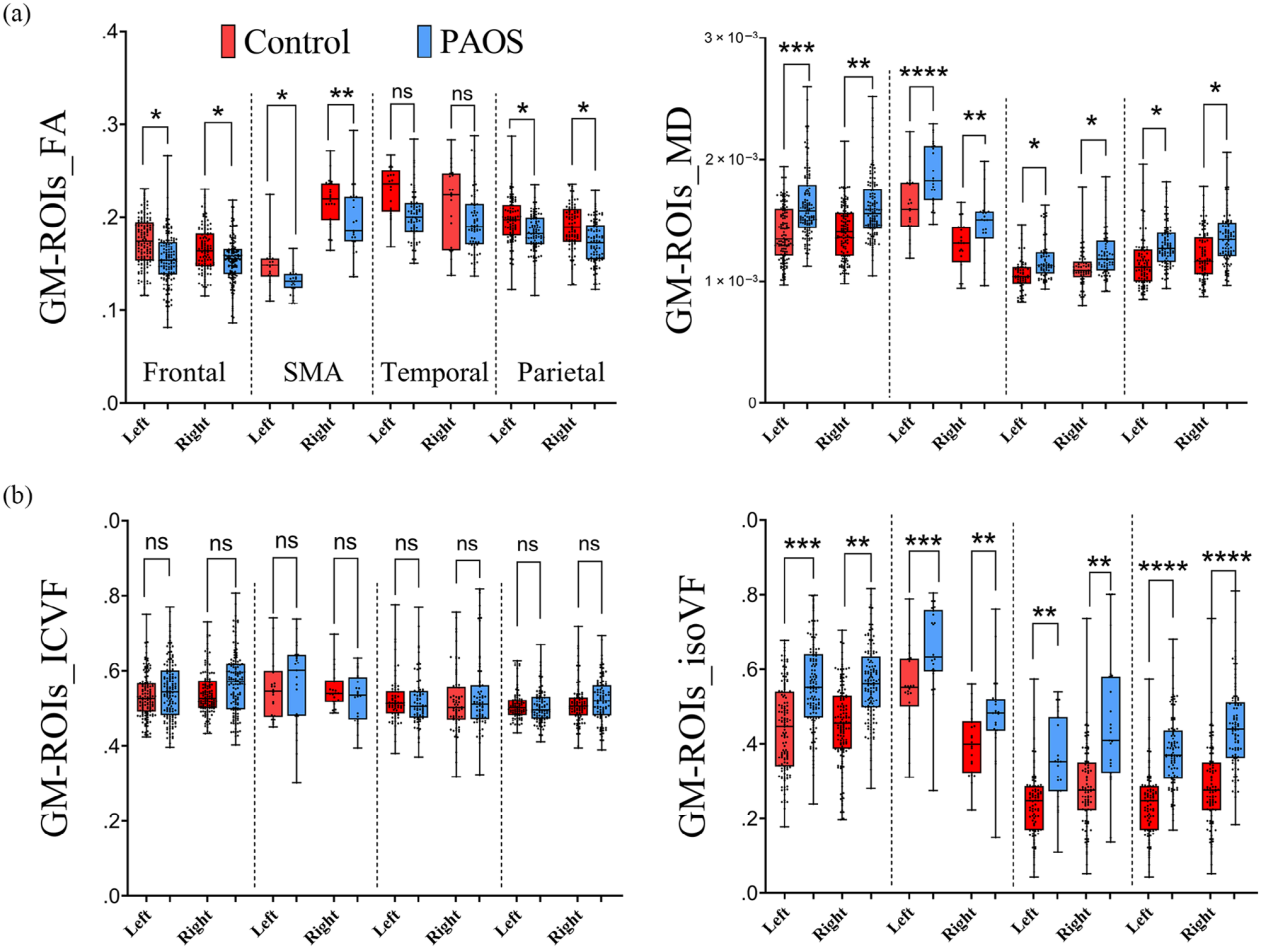
Evaluation of gray matter (GM) regions of interest (ROIs) by diffusion tensor imaging (DTI) and neurite orientation dispersion and density imaging (NODDI) algorithms. (A) Mean diffusivity (MD) analysis shows larger differences between groups across frontal, supplementary motor area (SMA), temporal, and parietal regions. (B) In the NODDI analysis, a large increase in isotropic volume fraction (IsoVF) was seen in the progressive apraxia of speech (PAOS) groups compared to the intracellular volume fraction (ICVF) analysis.

The DTI and NODDI findings measured across global WM and GM are shown in Figure 4. For DTI, a significant decrease in FA in PAOS compared to controls was observed in global WM (*p* < .02) and global GM (*p* < .02). Increase in MD was observed in global WM (*p* < .001) and GM (*p* < .005) (Figure 4A). NODDI outputs showed a significant decrease in ICVF global WM (*p* < .0001) and a large increase in IsoVF global GM (*p* < .0005) in PAOS compared to controls (Figure 4B). The DTI and NODDI findings in specific WM tracts are shown in Figure 5. All WM tracts showed significantly reduced FA and increased MD in PAOS compared to controls after FDR correction, except for FA of CC_Major, which showed a trend (*p* < .07) (Figure 5A). Similarly, all tracts showed reduced ICVF in PAOS compared to controls after FDR correction. However, for IsoVF, only differences in the CC_major, right FAT, left ILF, and bilateral anterior thalamus survived FDR correction (Figure 5B). The DTI and NODDI findings from GM regions are shown in Figure 6. Reduced FA was observed in PAOS compared to controls for the left and right frontal lobes and SMA after FDR correction. All GM regions showed increased MD in PAOS compared to controls after FDR correction, with the greatest differences in the left frontal lobe and SMA (Figure 6A). However, none of the GM regions showed differences between groups in terms of ICVF. Conversely, large differences between PAOS and controls were observed in the IsoVF metric in all GM regions investigated, including not only the frontal lobe and SMA but also in the temporal and parietal lobes (Figure 6B).

### 3.3 AUROC analysis

Evaluation of Areas Under de Receiver Operating Characteristics (AUROCs) for global WM and GM tracts showed DTI and NODDI modalities can discriminate microstructural differences between control and PAOS groups, with the highest AUC values in the global WM achieved with ICVF and in global GM achieved with MD (Table 2). Overall, MD showed high AUC values for discriminating PAOS from controls across nearly all WM tracts, with excellent discrimination (AUROCs >.95) for the CC_body fibers as well as the left and right ArcF, FAT, and Cstr_sup tracts. From NODDI, the AUC from ICVF was also high in the CC_body and left FAT, Thal_ant, and Cstr_sup tracts. The IsoVF did not perform well for the WM tracts but showed moderate AUCs along GM regions (Table 2).

**TABLE 2:** Comparative AUROC analysis between control and PAOS groups.

Control vs. PAOS [AUROC]		DTI		NODDI	
		FA	MD	ICVF	IsoVF
Global WM		.70	.71	**.84**	.67
Global GM		.71	.83	.60	.80
White matter tracts	CC_Minor	.79	.89	.75	.65
	CC_Body	**.95**	**.98**	**.94**	.77
	CC_Major	.68	.77	.68	.68
	ArcF_L	.84	**.95**	**.90**	.75
	ArcF_R	.80	**.96**	.88	.68
	FAT_L	**.97**	**.97**	**.97**	.67
	FAT_R	**.93**	**.99**	**.93**	.73
	ILF_L	.76	**.90**	.74	.79
	ILF_R	.70	.82	.72	.70
	Thal_ant_L	.77	.86	.86	.75
	Thal_ant_R	.78	.88	.80	.71
	Cstr_sup_L	.83	**.98**	**.95**	.60
	Cstr_sup_R	.77	**.96**	**.92**	.59
Gray matter regions	Frontal Lobe	.71	.73	.58	.78
	Temporal lobe	.62	.73	.57	.73
	Parietal lobe	.71	.75	.55	**.88**
	SMA	.76	.76	.78	**.74**

*Note*: Area under the receiving operator curves (AUROCs) comparing control and progressive apraxia of speech (PAOS) groups for DTI and NODDI parameters. Calculations across whole‐brain GM and WM, AUROC comparatives from significant WM tracts, and AUROCs from different cortical GM ROIs are presented. In bold, AUROC >.9 or comparatively significant.

Abbreviations: ant, anterior; ArcF, arcuate fasciculus; CC, corpus callosum; Cstr, corticostriatal; DTI, diffusion tensor imaging; FA, fractional anisotropy; FAT, frontal aslant tract; GM, gray matter; ICVF, intracellular volume fractions; ILF, inferior longitudinal fasciculus; IsoVF, isotropic volume fraction; L, left; MD, mean diffusivity; NODDI, neurite orientation dispersion and density imaging; R, right; SMA, supplementary motor area; sup, superior; Thal, thalamic; WM, white matter.

### 3.4 Clinical correlates of DTI and NODDI metrics

**FIGURE 7:**
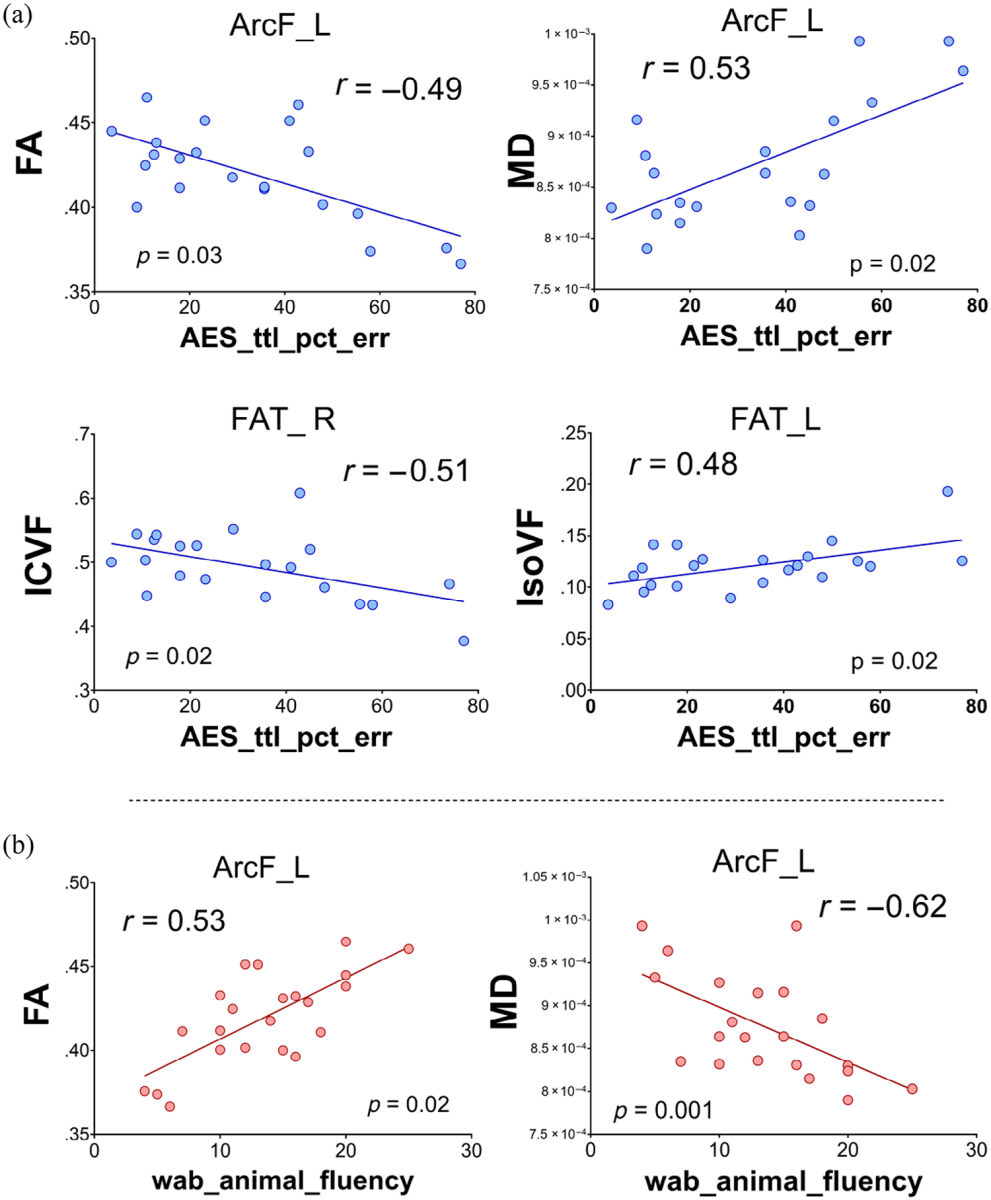
Correlative analysis of white matter (WM) tracts involved in language and clinical parameters. (A) Correlative analysis between diffusion tensor imaging (DTI) and neurite orientation dispersion and density imaging (NODDI) parameters shows significant correlations between articulatory error score total percentage of error (AES_tt_pct_err), with the left arcuate fasciculus (ArcF_L). Additional correlation can be obtained between NODDI parameters (intracellular volume fraction [ICVF] and isotropic volume fraction [IsoVF]) from the left frontal aslant tract (FAT_L) and AES_tt_pct_err scores. No large differences in rho were observed independently of diffusion magnetic resonance imaging (dMRI) techniques used (DTI vs. NODDI). (B) Additional significant correlations between Western Aphasia Battery (WAB) animal fluency (WAB_animal_fluency) were seen between fractional anisotropy (FA) (right) and mean diffusivity (MD) (left) from the ArcF_L and WAB_animal_fluency.

Spearman correlations were performed on DTI and NODDI parameters to examine the association between microstructural metrics and the severity of clinical symptoms, including the MoCA, WAB‐AQ, letter fluency, animal fluency, ASRS‐3, MDS‐UPDRS III, and WAB praxis (Table S1). We found that percentage of errors on the AES was associated with FA, MD, ICVF, and IsoVF in the left ArcF, FAT_L, and FAT_R (Figure 7A). AES also correlated with the DTI and NODDI metrics (Table S1). WAB animal fluency was significantly associated with FA and MD in the left arcuate fasciculus (ArcF_L) (Figure 7B).

### 3.5 Tract comparisons between patients with and without aphasia

We performed a secondary analysis assessing DTI and NODDI metrics in the PAOS patients with and without agrammatic aphasia (Table S2). Both groups showed similar patterns of abnormalities compared to controls, although only those with aphasia showed differences in FA and IsoVF in the ILF compared to controls. Very few differences were observed in direct comparisons between the patients with versus without aphasia, likely due to the small number of patients. However, the group with aphasia showed lower FA in the corpus callosum major (CC _Major) and greater IsoVF in the left thalamic anterior tract (Thal_ant_L) compared to those without aphasia.

## 4 DISCUSSION

In this study, we found that the application of diffusion models such as DTI and NODDI can detect microstructural abnormalities in WM and GM in patients with PAOS. Our findings point toward a complementary role of both diffusion techniques to detect different structural aspects of brain tissue degeneration and its relationship with brain dysfunction.

Multishell diffusion models have been previously used to assess structural changes and discriminability capabilities across different neurodegenerative diseases (Mitchell et al., 2019). From our evaluation of global WM microstructural changes in PAOS, ICVF provided the best discrimination of PAOS from controls and outperformed FA and MD. When assessing specific WM tracts, ICVF, MD, and FA performed comparably in differentiating PAOS from controls in the key tracts, such as the body of the corpus callosum and frontal aslant tracts, with all three showing excellent discrimination. The ICVF measure, which provides a marker of neuronal density, shows utility as a marker of WM integrity in PAOS and could add complementary information to DTI. However, MD outperformed ICVF and FA in the arcuate fasciculus, ILF, thalamic anterior radiation, and corticostriatal tracts, suggesting that MD may be more sensitive to subtle microstructural abnormalities in those tracts. The IsoVF metric did not perform well in any of the WM tracts suggesting little utility for this measure of extracellular free water in assessing WM integrity in PAOS. This implies that WM tracts are still highly ordered at this stage of the disease, and this water‐free compartment (IsoVF) is highly preserved. Studies in other neurodegenerative diseases have found mixed results regarding the comparative utility of DTI and NODDI metrics. One study found that DTI metrics, including FA, were more sensitive than NODDI measurements to early WM tract abnormalities in Alzheimer's disease (AD), although another found that NODDI measurements showed better relationships to tau pathology in AD (Weston et al., 2023).

We have previously shown that the SMA commissural fibers are affected in patients with PAOS (Valls Carbo et al., 2022). In the present study, with a separate cohort of PAOS patients, the analysis of WM tracts by co‐registration of DTI and NODDI reproduced changes in diffusivity in the body of the corpus callosum and demonstrated abnormalities in FA, MD, and ICVF. MD from the body of the corpus callosum provided excellent discrimination of PAOS from controls, with an AUROC of .98. The role of the ArcF in language and motor speech has been well‐established (Catani & Mesulam, 2008; Chenausky et al., 2020; Marchina et al., 2011). However, the involvement of this tract specifically in the context of PAOS is uncertain. Our previous findings suggest that the FAT is particularly associated with the presence of agrammatism in PAOS (Valls Carbo et al., 2022), with others finding associations with verbal fluency (Catani et al., 2013; La Corte et al., 2021). Similarly, a recent DTI study showed that WM structural volumes and FA can distinguish variants of primary progressive aphasia, with dorsal stream tracts, such as the superior longitudinal fasciculus, external capsule, and anterior and superior corona radiata, increasing the likelihood of the nonfluent/agrammatic variant diagnosis relative to the semantic variant (Bruffaerts et al., 2022; Keator et al., 2022). The ILF has also been implicated in language function in PPA (Mandelli et al., 2014). We found significant changes in the ILF, greater on the left, along with alterations in FA, as well as in MD and ICVF, although MD provided the best differentiation of PAOS from controls.

A previous longitudinal multimodal study by Sintini and colleagues combined structural MRI, rs‐fMRI, and [18 F] fluorodeoxyglucose (FDG)‐PET in patients with primary progressive aphasia and primary PAOS (Sintini et al., 2022). This study found that the rate of neurodegeneration correlated with functional connectivity to the premotor, motor, and frontal cortex as well as connectivity disruption related to the caudate and thalamus. Therefore, disrupted connectivity in the thalamocortical WM tracts seems to be implicated in neuropathology and functional features of PAOS. We found that the anterior thalamocortical tracts were impaired in PAOS. Similarly, the corticostriatal tracts were impaired supporting the role of basal ganglia connections and subcortical networks in speech and language production (Robles et al., 2005; Silveri, 2021). Other studies explored the link between striatal functions and language, proposing this subcortical structure as a central node for verbal executive network regulating the distributions of cognitive resources, such as verbal working memory and attention (Jacquemot & Bachoud‐Lévi, 2021). Disruptions of these WM tracts could also be related to the development of parkinsonism and other features of progressive supranuclear palsy and/or corticobasal syndrome in these PAOS patients (Seckin et al., 2020).

GM atrophy is a key feature in PAOS (Josephs et al., 2011), with involvement of the premotor cortex and spread over time into the prefrontal cortex, motor cortex, basal ganglia, and midbrain (Josephs et al., 2014). We observed global increased MD in the GM, particularly in the frontal lobes and SMA, consistent with the central role of the SMA in AOS (Duffy & Josephs, 2012). Increased IsoVF was also observed in the GM, with increases observed across most GM regions, with particularly striking abnormalities in the parietal lobe. This finding contrasts with the lack of IsoVF abnormalities in the WM tracts, suggesting the better utility of this measure of extracellular free water in interrogating the GM in PAOS. However, it is unclear why there were such striking IsoVF abnormalities in the parietal lobe. The parietal lobe is not typically affected early in PAOS, although it can become affected later in the disease (Tetzloff et al., 2018). It is possible that IsoVF is sensitive to very early changes in microstructure or that it captures anomalies in motor representation principally stored in parietal regions (Goldenberg, 2014; Kenneth, 2003), These results will need to be replicated in larger cohorts and assessed longitudinally to test this hypothesis. The ICVF metric did not identify any GM abnormalities in PAOS, suggesting that in contrast to IsoVF, it is a more appropriate and sensitive measure to interrogate the WM.

In terms of speech and language connectomics, we found that DTI and NODDI measures from the ArcF, FAT, and ILF were associated with the total percentage of errors that patients made repeating words and sentences in the AES, indicating a role of these tracts in the motor planning and programming difficulties associated with AOS. We also found significant correlations between ArcF_L, FAT_L, CC_body, and ILF_L for DTI (FA, MD) and verbal fluency performance. Previous studies have explained the relationship between WM integrity measured by DTI and verbal fluency based on age (Yeske et al., 2021). Nonetheless, age cannot account for our finding, as control and patients were matched for age. However, it is difficult to identify the precise nature of the relationship in this patient population given that the task requires cognitive planning and organization, lexical retrieval, and intact, rapid speech production.

Although some studies have assessed ODI (Reina et al., 2020), previous studies have demonstrated that ICVF is a preferable parameter to capture WM damage in the brain (Raghavan et al., 2022). In this study, we focus on the application of structural techniques to capture abnormalities at one time point. However, neurodegenerative events are dynamic processes in constant evolution. Therefore, the addition of functional imaging studies could give us a more comprehensive picture of how neurodegeneration in critical areas of language can relate to cognitive and behavioral features. Another limitation of this study is the relatively small number of patients with PAOS, although we did identified abnormalities that survived correction for multiple comparisons. Two subtypes of PAOS have been described, which may influence regional patterns of WM degeneration (Utianski et al., 2018), and this will need to be considered in future studies. Additional studies assessing MRI and neuropathological features will also help in the interpretation of how each diffusion metric relates to microstructural reconfiguration in brain tissue. Future studies will examine NODDI's longitudinal changes across diferent PAOS variants to determine the value of the analysed metrics as disease biomarkers.

## 5 CONCLUSIONS

The mixed application of DTI and NODDI is a beneficial approach to detecting microstructural anomalies in patients with PAOS. DTI parameters, such as FA and MD, and NODDI outcomes, such as ICVF and IsoVF, likely represent different aspects of brain tissue microstructure and show potential as novel disease biomarkers in this tauopathy. Although some parameters such as ICVF were more suitable to detect differences in global WM, others such as MD and IsoVF were more likely to detect GM anomalies in critical language‐related areas.

## AUTHOR CONTRIBUTIONS

**Rodolfo G. Gatto**: Conceptualization; investigation; methodology; visualization; formal analysis; writing—review and editing; writing—original draft; software. **Gabriela Meade**: Writing—review and editing; resources. **Joseph R. Duffy**: Writing—review and editing; resources. **Heather M. Clark**: Writing—review and editing; resources. **Rene L. Utianski**: Resources; writing—review and editing. **Hugo Botha**: Writing—review and editing; resources. **Mary M. Machulda**: Writing—review and editing; resources. **Keith A. Josephs**: Funding acquisition; supervision; writing—review and editing; conceptualization; resources; investigation. **Jennifer L. Whitwell**: Funding acquisition; supervision; writing—review and editing; conceptualization; resources; investigation.

### PEER REVIEW

The peer review history for this article is available at https://publons.com/publon/10.1002/brb3.3346.

## Supporting information

Supp Information

## Data Availability

The source data that support the findings of this study will be made available upon reasonable request.
