# Supplementary material for: Combined assessment of progressive apraxia of speech brain microstructure by diffusion tensor imaging tractography and multishell neurite orientation dispersion and density imaging
Source: Brain Behav. 2024 Jan 2;14(1):e3346. doi: 10.1002/brb3.3346 (PMC10761330; doi:10.1002/brb3.3346)
Supplement: Supplementary file 1 — Supp Information [file BRB3-14-e3346-s001.docx]

**Supplementary Table 1** - Spearman correlation coefficients between clinical scores and FA(a), MD (b), ICVF (c), and IsoVF (f) from WM tracts in PAOS.

| FA | MoCA | WAB AQ | ASRS 3 Total | AES_ttl_pct_err | UPDRS III+ | Apraxia (/60) | Letter_fluency_sum | WAB_animal_fluency |
| --- | --- | --- | --- | --- | --- | --- | --- | --- |
| CC_Minor | 0.04 | 0.18 | -0.30 | -0.29 | 0.01 | 0.19 | 0.38 | 0.38 |
| CC_Body | -0.03 | 0.03 | -0.02 | 0.01 | 0.06 | 0.22 | 0.43 | 0.36 |
| CC_Major | 0.02 | 0.25 | -0.04 | -0.19 | -0.40 | 0.52 | 0.43 | 0.07 |
| ArcF_L | 0.06 | 0.41 | -0.30 | **-0.49 (p<0.02)** | -0.16 | 0.47 | **0.58 (p<0.01)** | **0.59 (p<0.01)** |
| ArcF_R | 0.14 | 0.42 | -0.40 | **-0.54 (p<0.01)** | -0.05 | 0.39 | **0.47 (p<0.03)** | **0.50 (p<0.02)** |
| FAT_L | 0.32 | 0.29 | -0.21 | -0.43 | -0.14 | 0.39 | **0.52 (p<0.02)** | **0.55 (p<0.01)** |
| FAT_R | 0.25 | 0.29 | -0.22 | -0.35 | 0.11 | 0.27 | 0.42 | **0.46 (p<0.04)** |
| ILF_L | 0.10 | 0.31 | -0.09 | **-0.56 (p<0.01)** | -0.33 | 0.40 | **0.50 (p<0.02)** | **0.70 (p<0.01)** |
| ILF_R | 0.05 | 0.31 | -0.17 | **-0.47 (p<0.03)** | 0.12 | 0.32 | 0.41 | **0.47 (p<0.03)** |
| Thal_Ant_L | 0.27 | 0.41 | -0.11 | -0.38 | -0.23 | 0.42 | 0.37 | **0.63 (p<0.02)** |
| Thal_Ant_R | 0.00 | 0.15 | **-0.52 (p<0.01)** | -0.28 | -0.01 | 0.10 | 0.03 | 0.29 |
| CStr_Sup_L | 0.04 | 0.03 | -0.17 | -0.30 | -0.17 | -0.04 | 0.20 | **0.50 (p<0.02)** |
| CStr_Sup_R | 0.26 | 0.05 | -0.09 | -0.12 | 0.03 | 0.04 | 0.28 | 0.43 |

**a -** Spearman correlation coefficients between clinical scores and FA from WM tracts in PAOS.

**b -** Spearman correlation coefficients between clinical scores and MD from WM tracts in PAOS.

| MD | MoCA | WAB AQ | ASRS 3 Total | AES_ttl_pct_err | UPDRS III+ | Apraxia (/60) | Letter_fluency_sum | WAB_animal_fluency |
| --- | --- | --- | --- | --- | --- | --- | --- | --- |
| CC_Minor | -0.02 | -0.23 | 0.31 | **0.50 (p<0.04)** | -0.17 | -0.08 | -0.22 | **-0.49 (p<0.03)** |
| CC_Body | 0.04 | -0.09 | 0.17 | 0.10 | -0.05 | -0.03 | -0.18 | -0.36 |
| CC_Major | 0.15 | -0.10 | 0.13 | -0.04 | 0.10 | -0.25 | -0.16 | 0.15 |
| ArcF_L | 0.11 | -0.27 | 0.27 | **0.53 (p<0.02)** | 0.16 | -0.29 | **-0.47 (p<0.04)** | **-0.63 (p<0.01)** |
| ArcF_R | 0.03 | -0.32 | 0.39 | **0.55 (p<0.01)** | 0.23 | -0.22 | -0.37 | **-0.53 (p<0.02)** |
| FAT_L | -0.10 | -0.14 | -0.06 | 0.37 | 0.05 | -0.20 | -0.31 | -0.41 |
| FAT_R | -0.06 | -0.16 | 0.00 | 0.35 | -0.02 | -0.26 | -0.38 | -0.33 |
| ILF_L | 0.11 | -0.18 | 0.34 | 0.36 | -0.02 | -0.12 | -0.27 | -0.41 |
| ILF_R | -0.14 | -0.33 | 0.39 | 0.44 | 0.05 | -0.18 | -0.27 | -0.36 |
| Thal_Ant_L | -0.11 | -0.25 | 0.12 | **0.55 (p<0.01)** | 0.12 | -0.15 | -0.20 | **-0.49 (p<0.02)** |
| Thal_Ant_R | -0.16 | -0.18 | **0.46 (p<0.04)** | 0.41 | 0.01 | -0.12 | -0.15 | -0.15 |
| CStr_Sup_L | -0.05 | -0.16 | 0.01 | 0.31 | -0.02 | -0.15 | -0.31 | **-0.56 (p<0.01)** |
| CStr_Sup_R | -0.06 | -0.08 | 0.05 | 0.35 | -0.21 | -0.13 | -0.36 | **-0.46 (p<0.04)** |

| ICVF | MoCA | WAB AQ | ASRS 3 Total | AES_ttl_pct_err | UPDRS III+ | Apraxia (/60) | Letter_fluency_sum | WAB_animal_fluency |
| --- | --- | --- | --- | --- | --- | --- | --- | --- |
| CC_Minor | -0.20 | -0.13 | -0.14 | -0.27 | -0.07 | -0.20 | -0.09 | 0.07 |
| CC_Body | -0.21 | -0.03 | **-0.14 (p<0.04)** | -0.22 | -0.07 | -0.05 | 0.03 | 0.18 |
| CC_Major | -0.60 | -0.16 | 0.05 | -0.13 | -0.02 | -0.05 | 0.05 | -0.05 |
| ArcF_L | -0.11 | 0.23 | -0.31 | **-0.47 (p<0.04)** | -0.15 | 0.25 | 0.40 | 0.40 |
| ArcF_R | -0.12 | 0.02 | -0.33 | -0.31 | 0.01 | 0.10 | 0.26 | 0.20 |
| FAT_L | 0.12 | 0.11 | -0.10 | **-0.43 (p<0.02)** | 0.05 | 0.21 | 0.21 | 0.32 |
| FAT_R | 0.19 | 0.24 | -0.26 | -0.51 | -0.08 | 0.20 | 0.26 | 0.32 |
| ILF_L | -0.47 | -0.09 | -0.14 | -0.29 | -0.10 | -0.06 | 0.06 | 0.19 |
| ILF_R | **-**0.39 | 0.01 | -0.23 | -0.27 | -0.03 | 0.10 | 0.15 | 0.10 |
| Thal_Ant_L | -0.10 | 0.22 | **-0.34 (p<0.03)** | **-0.52 (p<0.03)** | -0.18 | 0.25 | 0.24 | 0.37 |
| Thal_Ant_R | -0.17 | 0.05 | -0.43 | -0.28 | -0.05 | 0.02 | 0.00 | 0.07 |
| CStr_Sup_L | -0.11 | 0.04 | -0.15 | -0.43 | -0.27 | 0.10 | 0.24 | 0.35 |
| CStr_Sup_R | 0.05 | 0.09 | -0.30 | -0.42 | 0.00 | 0.10 | 0.19 | 0.28 |

**c -** Spearman correlation coefficients between clinical scores and ICVF from WM tracts in PAOS.

**d-** Spearman correlation coefficients between clinical scores and isoVF from WM tracts in PAOS.

| IsoVF | MoCA | WAB AQ | ASRS 3 Total | AES_ttl_pct_err | UPDRSIII+ | Apraxia (/60) | Letter_fluency_sum | | WAB_animal_fluency |
| --- | --- | --- | --- | --- | --- | --- | --- | --- | --- |
| CC_Minor | -0.16 | -0.33 | 0.42 | 0.38 | -0.08 | -0.19 | -0.07 | -0.33 | |
| CC_Body | -0.14 | -0.16 | **0.44 (p<0.04)** | 0.19 | 0.11 | -0.08 | -0.13 | -0.16 | |
| CC_Major | 0.15 | 0.00 | 0.21 | 0.03 | 0.16 | -0.09 | -0.08 | 0.11 | |
| ArcF_L | -0.24 | -0.24 | 0.36 | **0.43 (p<0.03)** | -0.08 | -0.30 | -0.31 | -0.30 | |
| ArcF_R | -0.14 | -0.26 | 0.00 | 0.23 | 0.10 | -0.06 | -0.12 | -0.26 | |
| FAT_L | -0.32 | -0.37 | 0.39 | **0.49 (p<0.03)** | 0.18 | -0.23 | -0.15 | **-0.48 (p<0.02)** | |
| FAT_R | -0.21 | -0.35 | 0.37 | 0.42 | 0.09 | -0.14 | 0.03 | -0.32 | |
| ILF_L | -0.26 | -0.33 | 0.34 | 0.26 | -0.01 | -0.24 | -0.25 | **-0.22 (p<0.02)** | |
| ILF_R | **-0.46 (p<0.03)** | -0.22 | 0.07 | 0.29 | 0.15 | -0.02 | -0.07 | -0.32 | |
| Thal_Ant_L | -0.39 | -0.32 | 0.01 | **0.47 (p<0.03)** | 0.17 | -0.18 | -0.15 | -0.46 | |
| Thal_Ant_R | -0.36 | -0.15 | **0.47 (p<0.03)** | 0.35 | 0.17 | -0.01 | 0.16 | -0.02 | |
| CStr_Sup_L | -0.24 | -0.23 | 0.33 | 0.25 | -0.18 | -0.08 | -0.07 | -0.23 | |
| CStr_Sup_R | -0.25 | -0.29 | 0.42 | 0.34 | -0.17 | -0.11 | -0.07 | -0.23 | |

**Supplementary Table 2 -** Comparison between PPAOS and AOS+PAA vs DTI and NODDI parameters

|  |  | | FA | | | |  | MD | |  | ICVF | |  | IsoVF | |  |
| --- | --- | --- | --- | --- | --- | --- | --- | --- | --- | --- | --- | --- | --- | --- | --- | --- |
|  | |  | | AOS-PPA (n=14) | | PPAOS (n=9) | *p_val†* | AOS- PPA (n=14) | PPAOS  (n=9) | *p_val†* | AOS- PPA (n=14) | PPAOS (n=9) | *p_val†* | AOS- PPA (n=14) | PPAOS (n=9) | *p_val†* |
| global_gm | | | | 0.16* | | 0.16 | *0.89* | 0.0010* | 0.0010* | *0.84* | 0.53 | 0.52 | *0.75* | 0.47* | 0.48* | *0.67* |
| global_wm | | | | 0.30 | | 0.29* | *0.75* | 0.0014* | 0.0015* | *0.23* | 0.51* | 0.50* | *0.05* | 0.22* | 0.20 | *0.41* |
|  | |  | |  | |  |  |  |  |  |  |  |  |  |  |  |
| WM | | *CC_Minor* | | | 0.44* | 0.44* | *0.79* | 0.00093* | 0.00092* | *0.66* | 0.50* | 0.50* | *0.65* | 0.11* | 0.10 | *0.44* |
|  |  | *CC_Body* | | | 0.45* | 0.44 | *0.74* | 0.00095* | 0.00094* | *0.93* | 0.53* | 0.51* | *0.65* | 0.15* | 0.14 | *0.56* |
|  |  | *CC_Major* | | | 0.56* | 0.58 | **0.04** | 0.00095* | 0.00092 | *0.29* | 0.60 | 0.60 | *0.65* | 0.17* | 0.15 | *0.69* |
|  |  | *ArcF_L* | | | 0.41* | 0.43* | *0.47* | 0.00088* | 0.00087* | *0.59* | 0.53* | 0.53* | *0.74* | 0.10* | 0.10 | *0.37* |
|  |  | *ArcF_R* | | | 0.40* | 0.41* | *0.51* | 0.00088* | 0.00085* | *0.39* | 0.53* | 0.53* | *0.95* | 0.11* | 0.09 | *0.07* |
|  |  | *FAT_L* | | | 0.32* | 0.33* | *0.51* | 0.00096* | 0.00095* | *0.93* | 0.48* | 0.48* | *0.90* | 0.13* | 0.11 | *0.07* |
|  |  | *FAT_R* | | | 0.32* | 0.33* | *0.39* | 0.00095* | 0.00093* | *0.96* | 0.49* | 0.50* | *0.47* | 0.12* | 0.11 | *0.11* |
|  |  | *ILF_L* | | | 0.42* | 0.44 | *0.08* | 0.00089* | 0.00088* | *0.79* | 0.51* | 0.49* | *0.43* | 0.10* | 0.09 | *0.18* |
|  |  | *ILF_R* | | | 0.43* | 0.44 | *0.51* | 0.00088* | 0.00087* | *0.39* | 0.50 | 0.49* | *0.47* | 0.09* | 0.08 | *0.20* |
|  |  | *Thal_Ant_L* | | | 0.37* | 0.38 | *0.16* | 0.00096* | 0.00089* | *0.11* | 0.50* | 0.50* | *0.69* | 0.12* | 0.09 | **0.01** |
|  |  | *Thal_Ant_R* | | | 0.38* | 0.38* | *0.56* | 0.00093* | 0.00091* | *0.54* | 0.51* | 0.50* | *0.51* | 0.11* | 0.10 | *0.18* |
|  |  | *CStr_Sup_L* | | | 0.38* | 0.38* | *0.84* | 0.00089* | 0.00088* | *0.77* | 0.52* | 0.52* | *0.95* | 0.11 | 0.10 | *0.34* |
|  |  | *CStr_Sup_R* | | | 0.38* | 0.38* | *0.74* | 0.00089* | 0.00089* | *0.88* | 0.53* | 0.53* | *0.95* | 0.11 | 0.10 | *0.40* |
|  | |  | | |  |  |  |  |  |  |  |  |  |  |  |  |
| GM | | *Frontal_L* | | | 0.15* | 0.15* | *0.68* | 0.00164* | 0.00167* | *0.59* | 0.57 | 0.52 | *0.04* | 0.56* | 0.56* | *0.72* |
|  |  | *Frontal _R* | | | 0.16 | 0.16 | *0.43* | 0.00011* | 0.000158* | *0.05* | 0.58 | 0.53 | *0.05* | 0.53* | 0.54* | *0.82* |
|  |  | *Temporal _L* | | | 0.20* | 0.21 | *0.06* | 0.00119* | 0.00117* | *0.55* | 0.54 | 0.49 | *0.01* | 0.33* | 0.29* | *0.09* |
|  |  | *Temporal _R* | | | 0.19 | 0.20 | *0.91* | 0.00121* | 0.00127* | *0.43* | 0.55 | 0.51 | *0.25* | 0.37* | 0.36* | *0.72* |
|  |  | *Parietal_L* | | | 0.18 | 0.19 | *0.63* | 0.00131* | 0.00128* | *0.91* | 0.52 | 0.49 | *0.18* | 0.38* | 0.37* | *0.72* |
|  |  | *Parietal_L* | | | 0.17* | 0.17 | *0.87* | 0.00136* | 0.00134* | *0.72* | 0.54 | 0.50 | *0.16* | 0.44* | 0.44* | *0.63* |

**Footnote: ***Values significantly different from controls, p<0.05; † p values compared PPAOS and AOS-PAA. *In bold significant p values between AOS-PAA and PAOS groups comparisons.* All p values from nonparametric Mann Whitney test.
